# Consequences of tuberculosis among asylum seekers for health care workers in Germany

**DOI:** 10.1186/s12995-016-0093-x

**Published:** 2016-02-16

**Authors:** Roland Diel, Robert Loddenkemper, Albert Nienhaus

**Affiliations:** Institute for Epidemiology, University Medical Hospital Schleswig-Holstein, Airway Research Center North (ARCN), Niemannsweg 11, 24015 Kiel, Germany; German Central Committee against Tuberculosis, Berlin, Germany; Institute for Health Services Research in Dermatology and Nursing, University Medical Center, Hamburg-Eppendorf, Germany; Institution for Statutory Accident Insurance and Prevention in the Health and Welfare Services (BGW), Hamburg, Germany

**Keywords:** Health care workers, Asylum seekers, Tuberculosis, MDR-TB

## Abstract

**Background:**

Immigrants have been contributing to the incidence of tuberculosis (TB) in Germany for many years. The current wave of migration of asylum seekers to Germany may increase that figure. Healthcare workers (HCW) who look after refugees not only in hospitals and medical practices but also in aid projects may be exposed to cases of TB.

**Methods:**

The incremental TB cases arising from imported TB as well as from TB cases that developed later in refugees were calculated in a Markov model over a period of 5 years. Infectious and non-infectious susceptible TB and multidrug-resistant TB (MDR-TB) cases were determined separately. In addition, the total amount of latent TB in contact persons and the risk of infection by HCW were estimated. Due to uncertainty of future refugee flows to Europe, different scenarios were considered in univariate and multivariate sensitivity analysis.

**Results:**

Assuming a decrease in immigration by half each year to the bottom line of 2014, and in light of the current number of 800,000 asylum seekers, we calculated an additional 10,090 TB cases by the end of the fifth year (5976 cases of infectious pulmonary TB and 143 cases of pulmonary MDR-TB). In case of an unchanging influx of asylum seekers over the 5-year period, 19,031 TB cases would arise, 377 of which infectious MDR-TB. Eighty -seven ensuing TB cases would develop in HCW in the same period, 3 of which MDR-TB cases.

**Conclusions:**

Although the total number of TB cases in HCW expected to ensue from the current influx of asylum seekers is rather small, the 3 MDR-TB cases we calculated have to be taken seriously. We consider it essential to increase awareness of protective measures such as respiratory masks and, in the event of documented exposure, of supply-oriented occupational health screening.

## Background

Immigrants have been contributing toward the incidence of tuberculosis (TB) in Germany for many years. TB incidence is now more than thirteen times higher among residents who are foreign nationals than among persons born here [1], and in 2014 more than half (62.4 %) of all TB patients were foreign born. Not only is there a comparatively high frequency among first-generation immigrants, but a cross-sectional study conducted in 2012 in Berlin found TB incidence in the second generation to be at least twice as high as that found in Germans born of native parents. (10.4 versus 4.6 TB cases per 100,000 residents) [2]. At present, more than 1000 people arrive in Germany every day (a total of 54,877 refugees only in October 2015 (http://www.bamf.de/DE/Infothek/Aktuelles/aktuelles-node.html)), most of them from Syria, Afghanistan, Iraq, Eritrea and different Balkan states; no end to the influx is foreseen. The prevalence of active TB and consequently latent TB infection (LTBI) is known to be quite high in most of these countries, markedly different from the low rates found in Germany.

Many of these asylum seekers arrive after a dangerous journey on which they have suffered hunger, exposure, overcrowded accommodation and intolerable sanitary conditions, all of which place LTBI cases at elevated risk for progression to active, transmissible TB disease. Refugees are cared for in Germany by healthcare workers not only as TB patients in hospitals and medical practices but also from aid agencies. Transmission of TB from immigrants to persons born in Germany is statistically rare [3], but with the expected arrival of TB disease with refugees the incidence of TB infections leading to illness can be expected to rise among healthcare workers (HCW), who are often the first point of contact with immigrants and most exposed. It is therefore of special interest to accident insurers to understand how an increase in TB infections via immigrants may have impact on the number of TB cases among HCW in the future. This study models the possible development over the next 5 years and discusses the associated consequences.

## Methods

- Statutory examination of asylum seekers at the place of residence

Under Section 36 (4) of the German Infectious Diseases Law (Infektions-schutzgesetz, IfSG), persons who are to be admitted to shared accommodation sites for refugees or asylum seekers^1^ must produce a medical certificate stating that there are no signs of potentially infectious pulmonary TB. In the case of individuals aged 15 or older, with the exception of pregnant women, the certificate must be based on a chest X-ray [4].

Neither the number of chest X-ray examinations done in the months since the flood of refugees began nor the number and country of birth of persons whose X-rays show signs of TB are known, as it is not mandatory for German health authorities, reception centres performing the X-rays or contractual practices to record these data. Although Section 11 Paragraph 8 of the IfSG stipulates that the relevant state authorities must be notified of the citizenship and nationality of any person with the disease, the wording “admission to shared accommodation” does not distinguish between refugee accommodation, hostels for homeless persons, or care homes, and there is no separate “asylum seekers” category.

b) The risk of TB occurrence among asylum seekers over time

**Fig. 1:**
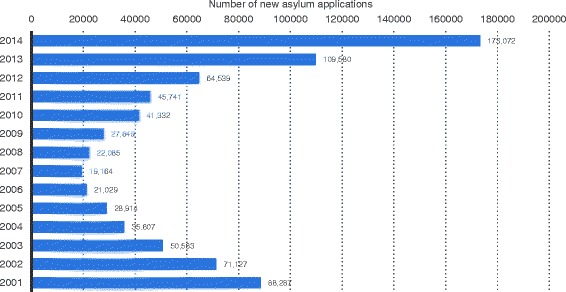
New applications for asylum in Germany 2001–2014. Source: modified according to Statistica 2015 and [5]

173,072 applications for asylum were lodged in Germany in 2014 [5] (see Fig. 1).

In the same year, 409 persons were recorded as having been diagnosed with TB upon admission to shared accommodation for asylum seekers [6]. Taking this figure as the denominator, there were 236 TB cases per 100,000 new applications, a ratio of 0.236 %.

This ratio seems to have changed hardly in recent years. The study by Diel et al. [3], until now the only one to have examined the results of screening in accordance with Section 36 (4) of the IfSG – carried out on asylum seekers in Hamburg over a long period (from 1997 to 2003) – found a ratio of 0.25 %. In the absence of other valid surveys and for the sake of simplicity, for our modelling we took this figure (probability of TB ascertained on entry screening [pSick_Entry], see Table 1) as the annual probability in the base year analysis. Arithmetically, with an assumed number of 800,000 refugees per year throughout Germany, this results in 2000 cases of TB diagnosed on at- entry screening.

**Table 1:** Input variables for the dynamic disease model

Category of variable	Name of variable	Basic value	Reference
Number of refugees arriving per year	T_refugees	Decreasing number from 1st to 4th year, then plateau	Federal Ministry of the Interior estimate of 19 August 2015 (http://www.bamf.de/DE/Infothek/Aktuelles/aktuelles-node.html)
Probability of deportation per year	pExit	0.0628	Calculated from data in [13] and (https://www.tagesschau.de/inland/abschiebungen-103.html)
Probability of TB ascertained on entry screening	pSick_Entry	0.0025	Diel et al. [3]
Probability of infectious pulmonary TB	pInfect_TB	0.6121	RKI 2015 [8]
Probability of later development of TB in refugees (up to 5 years after entry)	T_active	Decreasing probability from 1st to 5th year	Calculated from data in Diel et al. [3]
Probability of MDR-TB in cases of infectious pulmonary TB	pInfect_MDR_TB	0.0324	Calculated from RKI data 2015 [9]
Probability of MDR-TB in cases of non-infectious TB	pMDR_TB	0.010	RKI 2015 [9]

A recent meta-analysis [7], summarizing the findings of 22 studies that reported cases of active pulmonary tuberculosis among 2,620,739 screened refugees, asylum seekers and regular immigrants, even reported a yield for pulmonary TB of 3.5 cases (95 % CI 2.9.-4.1) per 1000 persons screened.

Diel et al. [3] also found that asylum seekers who stayed in the country ran a considerable risk of suffering TB disease in the four subsequent years (1.64 %% in the first year, 1.56 %% in the second, 0.99 %% and 0.57 %% in the third and fourth, and 0.41 %% in the fifth year). These probabilities of contracting TB within 5 years [T_active] were used for modelling.

c) Infectious tuberculosis

In 2013, 76.9 % of all notified TB cases in Germany (3298/4287) were pulmonary tuberculosis [8], of which 79.6 % (2624/3298), that is, 61.21 % of the total, were open (confirmed by culture) (probability of infectious pulmonary TB [pInfect_TB]). Of these, 45 % showed positive in sputum smears (1181/2624) and 55 % (1443/2624) negative. Open pulmonary tuberculosis accounted for 85 of the 102 notified multi-drug-resistant TB (MDR-TB) cases, that is, 83.3 %. Of these, 63.5 % (54/85) showed positive in sputum smears [9]. One can therefore assume that 3.2 % of all cases of open pulmonary TB are open pulmonary MDR-TB (probability of MDR-TB in cases of infectious pulmonary TB [pInfect_MDR_TB]). As a consequence there will be secondary cases among contact persons.

d) Non-infectious tuberculosis

The number of cases of non-infectious tuberculosis, regardless of organ manifestation, was calculated from the difference between the total number of cases and the number of open pulmonary tuberculosis infections (1-pInfect_TB). Seventeen out of 1663 cases of non-open tuberculosis in 2013, that is, 1 %, were cases of MDR-TB (probability of MDR-TB in cases of non-infectious TB [pMDR_TB]) [8].

e) Contact persons and probability of progression to tuberculosis

The average number of contact persons that a patient with open pulmonary TB will infect cannot be precisely predicted, as the literature shows it to be a highly situation-dependent phenomenon. If one assumes that on average a sputum-smear-positive index case infects five contact persons [10] and a sputum-smear-negative case one [11], the weighted mean value amounts to three infected contact persons per case of open pulmonary TB taking into account the above-mentioned distribution of sputum-smear positive and sputum-smear negative pulmonary TB that was also found in previous years [12].

f) Deportations of asylum seekers who have entered the country

When considering how the influx of asylum seekers may effect TB incidence in the coming years, one needs to take into account the proportion of new applicants who are deported. Officially, in 2014, of the 128,911 asylum applications on which a ruling was given, 68.6 % were rejected (88,348 in all) [13], but, in fact, only 10,884 individuals were deported. Applying this historic percentage of applicants deported to the number of new applications, the percentage of those leaving the pool of persons who may develop tuberculosis after 1 year is just 6.28 % (10,884 out of the 173,072 applications for asylum in 2014).

According to media reports (https://www.tagesschau.de/inland/abschiebungen-103.html), 8178 rejected asylum seekers were deported in the first half of 2015. Given the marked year-on-year increase in the number of refugees, it is not anticipated that the percentage will be higher in 2015.

### Model structure

**Fig. 2:**
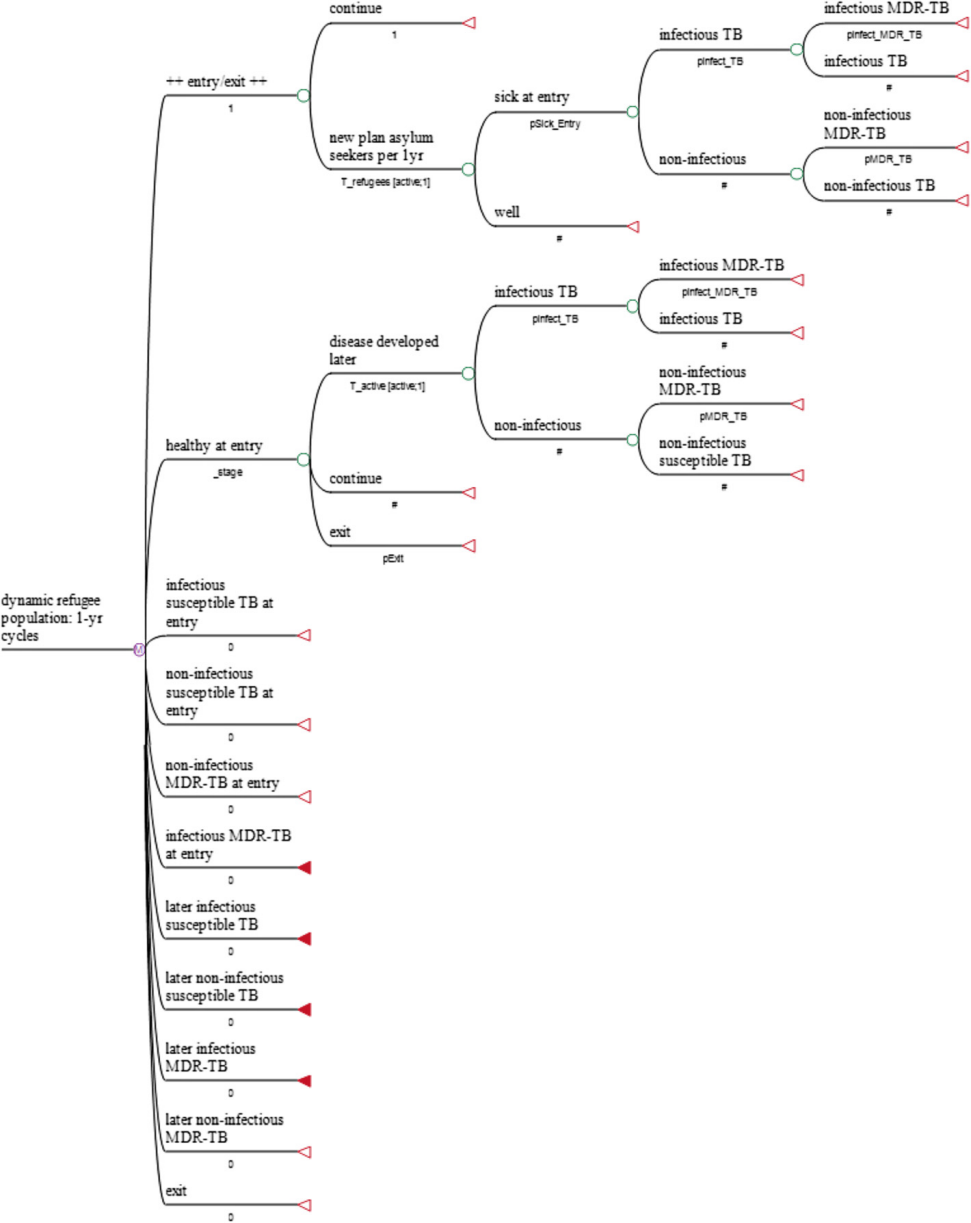
Markov decision tree (skeleton). New applications for asylum in Germany 2001–2014

We used TreeAge software (Version 2015) to develop a dynamic Markov decision tree, in which the duration of each cycle is 1 year (Fig. 2). To do this we had to make several assumptions on the following key parameters:

In the base model, the decision tree assumes that, in 2015, 800,000 asylum seekers arriving in Germany will be X-rayed in accordance with Section 36 (4) of the IfSG, and that 6.28 % of the asylum seekers arriving between 2015 and 2019 will be deported; the latter can therefore not develop TB in Germany in the years ahead. Indeed, currently up to 1,500,000 refugees are expected to arrive in Germany in 2015. As many, though, may fail to register and can therefore not be screened, our estimate of 800,000 refugees is deliberatively conservative. It will further be assumed that all asylum seekers who develop the disease will remain in Germany

In the absence of reliable trending data on immigration of non-EU citizens into Germany, we made a further conservative estimate and assumed a year-on-year halving of numbers for 2016 and 2017, i.e. that there will be 400,000 refugees in 2016 and 200,000 in 2017, and that in 2018 and 2019 the figure will remain at the 2014 level (173,072 asylum applications). Given the current increase in the number of refugees from Afghanistan, a high-incidence country, and that more than 4 million Syrians have had to flee to neighbouring countries whilst around 7.6 million Syrians have been displaced within Syria (http://www.unhcr.de/home/artikel/b0843b46d8393e8e4bf87511ff1c7b1c/zahl-der-syrien-fluechtlinge-uebersteigt-4-millionen.html), we assumed for the sensitivity analysis a worst-case scenario of an unchanging influx of registered 800,000 refugees in each of the 5 years.

According to our base probability described above, asylum seekers present with TB at the rate of 2.5 per 1000 persons initially X-rayed. Those refugees who do not have the disease and are not deported in subsequent years may go on to develop TB in line with the probability for each year as stated in the Table (T_active). The number of TB cases found on X-ray examination of new immigrants and those falling sick later are added up over 5 years. Table 1 provides an overview on all input variables finally programmed.

Due to uncertainty over a) the number of asylum seekers per year, b) the probability of discovering TB by at-entry screening, and c) the probabilities of contracting the disease in the next few years, we conducted several univariate and multivariate sensitivity analyses for these parameters, both singly and in combination. In one analysis, the probability of “pSick_Entry” was halved, as were all probabilities in the Table for “T_active”. As a worst-case scenario, the number of 800,000 immigrants per year was not reduced in the sensitivity analysis, but held constant for all 5 years. In the multivariate sensitivity analyses we either simultaneously halved the probabilities for “pSick_entry” and “T_active” and also halved the yearly numbers of immigrants or left the number of immigrants constant at 800,000 per year.

## Results

The results of our calculations are in Table 2. There are three different scenarios taken into consideration: The base analysis (scenario 1) shows 10,090 cases of tuberculosis by the end of the fifth year, of which 5976 are cases of infectious drug-susceptible pulmonary TB and 143 are cases of infectious pulmonary MDR-TB. If one assumes an unchanging influx of asylum seekers over the 5-year period, there are 19,031 TB cases, of which 11,272 have infectious drug-susceptible pulmonary TB and 377 have infectious pulmonary MDR-TB (worst-case scenario). The most favourable scenario, which assumes that TB cases found on chest X-ray screening are halved and that the probability of developing the disease over time is also halved, results in 5051 TB cases among immigrants, with 2991 cases of infectious drug-susceptible pulmonary TB and 100 cases of pulmonary MDR-TB (best-case scenario).

**Table 2:** Results and sensitivity analyses

a) Base case										
Year	Healthy at entry	Infectious TB at entry	Non-infectious TB at entry	Non-infectious MDR-TB at entry	Infectious MDR-TB at entry	Later infectious TB	Later non-infectious TB	Later infectious MDR-TB	Later non-infectious MDR-TB	Exit
1	798,000.00	1184.54	768.04	7.76	39.66	775.11	251.60	12.99	2.54	0.00
2	1,145,576.88	1776.80	1152.06	11.64	59.50	1833.55	595.37	30.75	6.01	50,177.20
3	1,271,347.55	2072.94	1344.07	13.58	69.41	2575.24	836.41	43.19	8.45	122,250.73
4	1,362,893.97	2329.20	1510.23	15.25	77.99	3038.57	987.03	50.97	9.97	202,286.12
5	1,449,161.25	2585.46	1676.39	16.93	86.57	3390.47	1101.44	56.88	11.13	288,111.21
10,090 cases (5976 infectious drug-susceptible TB cases, 143 infectious MDR-TB cases)										
b) 800,000 asylum seekers every year										
Year	Healthy at entry	Infectious TB at entry	Non-infectious TB at entry	Non-infectious MDR-TB at entry	Infectious MDR-TB at entry	Later infectious TB	Later non-infectious TB	Later infectious MDR-TB	Later non-infectious MDR-TB	Exit
1	798,000.00	1184.54	768.04	7.76	39.66	775.11	502.58	25.95	5.08	0.00
2	154,4576,88	2369.07	1536.08	15.52	79.33	2202.21	1427.89	73.74	14.42	5,0114.40
3	2,243,167.91	3553.61	2304.13	2327	118.99	3510.83	2276.39	117.56	22.99	147,113.83
4	2,898,087.45	4738.14	3072.17	31.03	158.66	4496.07	2915.21	150.55	29.45	287,984.77
5	3,512,424.05	5922.68	3840.21	38.79	198.32	5348.99	3468.24	179.11	35.03	469,984.66
19,031 cases (11,272 infectious drug-susceptible TB cases, 377 infectious MDR-TB cases)										
c) Halved TB prevalence at chest X-ray screening and 800,000 asylum seekers every year										
Year	Healthy at entry	Infectious TB at entry	Non-infectious TB at entry	Non-infectious MDR-TB at entry	Infectious MDR-TB at entry	Later infectious TB	Later non-infectious TB	Later infectious MDR-TB	Later non-infectious MDR-TB	Exit
1	799,000.00	592.27	384.02	3.88	19.83	776.08	502.58	25.95	5.08	0.00
2	1,546,512.44	1184.54	768.04	7.76	39.66	2204.97	1427.89	73.74	14.42	50,114.40
3	2,245,978.90	1776.80	1152.06	11.64	59.50	3515.23	2276.39	117.56	22.99	147,113.83
4	2,901,719.14	2369.07	1536.08	15.52	79.33	4,501,71	2915.21	150.55	29.45	287,984.77
5	3,516,825.59	2961.34	1920.11	19.40	99.16	5355.70	3468.24	179.11	35.03	469,984.66
14,043 cases (8317 infectious drug-susceptible TB cases, 278 infectious MDR-TB cases)										
d) Halved TB prevalence at chest X-ray screening, halved probabilities of developing TB in the following 5 years and 800,000 asylum seekers every year										
Year	Healthy at entry	Infectious TB at entry	Non-infectious TB at entry	Non-infectious MDR-TB at entry	Infectious MDR-TB at entry	Later infectious TB	Later non-infectious TB	Later infectious MDR-TB	Later non-infectious MDR-TB	Exit
1	7,990,00.00	592.27	384.02	3.88	19.83	388.04	503.21	25.99	5.08	0.00
2	1,547,167.62	1184.54	768.04	7.76	39.66	1102.79	1429.68	73.83	14.44	50,177.20
3	2,247,798.70	1776.80	1152.06	11.64	59.50	1758.45	2279.24	117.71	23.02	147,298.18
4	2,904,529.90	2369.07	1536.08	15.52	79.33	2252.17	2918.87	150.74	29.48	288,345.66
5	3,520,291.83	2961.34	1920.11	19.40	99.16	2679.58	3472.58	179.34	35.08	470,573.62
9524 cases (5641 infectious drug-susceptible TB cases, 279 infectious MDR-TB)										
e) Halved TB prevalence at chest X-ray screening and halved probabilities of developing TB in the following 5 years										
Year	Healthy at entry	Infectious TB at entry	Non-infectious TB at entry	Non-infectious MDR-TB at entry	Infectious MDR-TB at entry	Later infectious TB	Later non-infectious TB	Later infectious MDR-TB	Later non-infectious MDR-TB	Exit
1	7,990,00.00	592.27	384.02	3.88	19.83	388.04	251.60	12.99	2.54	0.00
2	1,147,667.62	888.40	576.03	5.82	29.75	918.23	595.37	30.75	60.10	50,177.20
3	1,274,448.91	1036.47	672.04	6.79	34.71	1289.98	836.41	43.19	8.45	122,250.73
4	1,366,641.51	1164.60	755.12	7.63	39.00	1522.28	987.03	50.97	9.97	202,286.12
5	1,453,279.86	1292.73	838.19	8.47	43.29	1698.73	1101.44	56.88	11.13	288,111.21
5051 cases (2991 infectious drug-susceptible TB cases, 100 infectious MDR-TB cases)										

In line with the results of the base analysis, this would mean an average number of 6119 × 3 infected contact persons (18,529). Unfortunately there are no officially reported data, but with a conservative estimate, assuming that only one in ten contact persons is a HCW, arrives at 1853 infected healthcare workers. With a risk of 5 % of developing the disease in the first 2 years after a fresh infection [12], one can expect 84 consequential cases of drug-susceptible active TB and 3 cases of MDR-TB by the end of the fifth year. In our best- case scenario still 42 cases of drug-susceptible TB and 1 case of MDR-TB would occcur, whereas the worst-case scenario results in 156 and 5 drug-susceptible TB and MDR-TB cases, respectively.

## Discussion

Asylum seekers can be assumed to have a higher risk of TB, for several reasons:

1. They are often from countries with a high prevalence of TB [14].
2. A high proportion is of age groups that are particularly affected in the country of origin (in particular young adults aged between 25 and 34) [15].
3. The acute psychosocial and/or physical strains of fleeing their home country are among the factors known to promote reactivation of a previously acquired LTBI [7].
4. They may have been exposed to TB during the various stages of a journey that has often taken months or years.

**Fig. 3:**
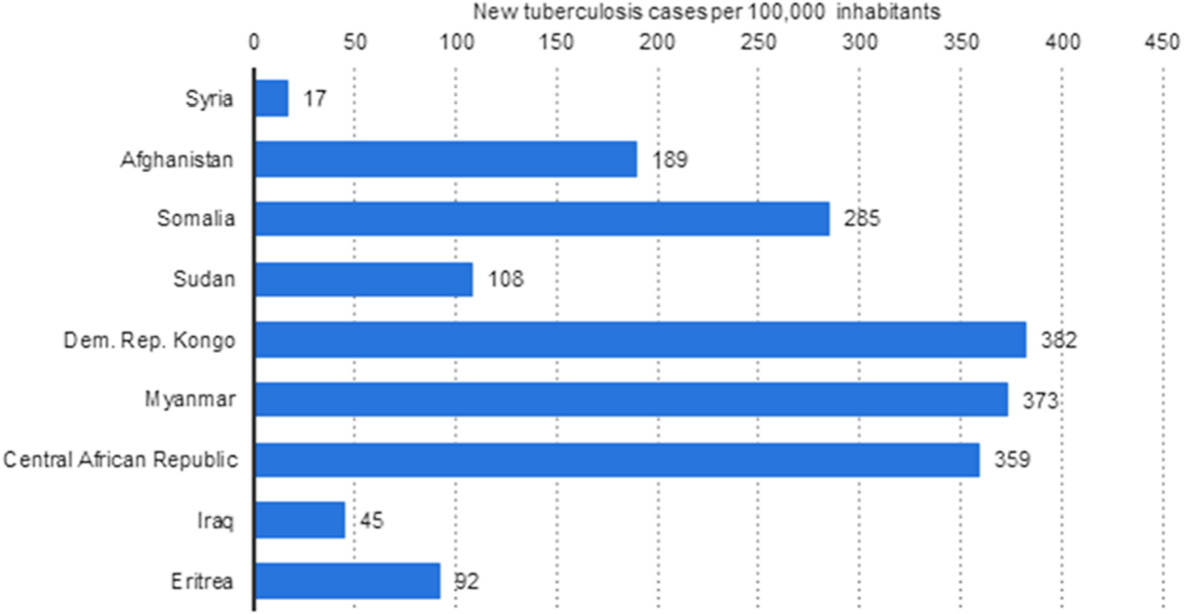
TB incidence in asylum seekers’ nine main countries of origin. Source: modified according to [19] and [20]

Eight of the nine main countries of origin of immigrants worldwide are countries with a high TB incidence > 20 per 100.000 population (Fig. 3).

For many years, there was a declining trend in new cases of TB notified to the German Robert Koch Institute (RKI), but this decline had ceased even before the current rise in refugee numbers [16]: In 2014, 4448 cases were notified and the incidence significantly increased with 5.6/100,000 [1], as against 4210 cases of TB and an incidence of 5.2/100,000 in 2012 [8].

In 2013, the Institute for Statutory Accident Insurance and Prevention in the Health and Welfare Services (BGW) alone received notification of 160 cases of active TB in HCW. That is 3.7 % of the 4318 cases reported to the RKI. A further 383 notifications were related to detected latent infections. However, one can assume that the actual number of TB cases to be currently expected among healthcare workers in Germany is higher because the TB cases notified to other accident insurers have not been taken into account.

Without any doubt, the risk of progression to TB is highest in the first year following the arrival of an immigrant in Germany and decreases as time goes on [3]. Nonetheless, the risk after several years of residency should certainly not be ignored and is therefore included in our calculation: Marx et al. [2] observed a significantly high number of TB cases among Berlin migrants in the first 9 years after immigration. While 28.4 % of cases occurred in the first year, the median period of latency between immigration and notification of TB was 8 years.

An earlier Danish study [17] on Somali asylum seekers found that the initially high rate of TB (3.0 % of all Somali immigrants) declined only gradually and that after 7 years 9.5 % of Somalis had suffered TB disease in their new country.

## Conclusions

Although the number of consequential TB cases in HCW to be expected in our base analysis is rather small, having been calculated at 87 cases in the next 5 years, one still has to expect 3 complicated MDR-TB cases requiring protracted treatment. It is therefore essential to increase awareness of protective measures such as respiratory masks and, in the event of documented exposure, of supply-oriented occupational health screening [18].

## Footnotes

1 The term “asylum seekers” covers asylum applicants, refugees who are unaccompanied minors, other refugees and tolerated persons.
